# Clustered Intracellular Salmonella enterica Serovar Typhimurium Blocks Host Cell Cytokinesis

**DOI:** 10.1128/IAI.00062-16

**Published:** 2016-06-23

**Authors:** António J. M. Santos, Charlotte H. Durkin, Sophie Helaine, Emmanuel Boucrot, David W. Holden

**Affiliations:** a Section of Microbiology, Medical Research Council Centre for Molecular Bacteriology and Infection, Imperial College London, London, United Kingdom; b Graduate Program in Areas of Basic and Applied Biology (GABBA), University of Porto, Porto, Portugal; c Institute of Structural and Molecular Biology, Division of Biosciences, University College London, London, United Kingdom; The University of Massachusetts Medical School

## Abstract

Several bacterial pathogens and viruses interfere with the cell cycle of their host cells to enhance virulence. This is especially apparent in bacteria that colonize the gut epithelium, where inhibition of the cell cycle of infected cells enhances the intestinal colonization. We found that intracellular Salmonella enterica serovar Typhimurium induced the binucleation of a large proportion of epithelial cells by 14 h postinvasion and that the effect was dependent on an intact Salmonella pathogenicity island 2 (SPI-2) type 3 secretion system. The SPI-2 effectors SseF and SseG were required to induce binucleation. SseF and SseG are known to maintain microcolonies of Salmonella-containing vacuoles close to the microtubule organizing center of infected epithelial cells. During host cell division, these clustered microcolonies prevented the correct localization of members of the chromosomal passenger complex and mitotic kinesin-like protein 1 and consequently prevented cytokinesis. Tetraploidy, arising from a cytokinesis defect, is known to have a deleterious effect on subsequent cell divisions, resulting in either chromosomal instabilities or cell cycle arrest. In infected mice, proliferation of small intestinal epithelial cells was compromised in an SseF/SseG-dependent manner, suggesting that cytokinesis failure caused by *S*. Typhimurium delays epithelial cell turnover in the intestine.

## INTRODUCTION

*S*almonella enterica serovar Typhimurium is a causative agent of self-limiting gastroenteritis. Following oral ingestion, *S*. Typhimurium invades and colonizes the intestinal epithelium, Peyer's patches, and mesenteric lymph nodes (1). Salmonella virulence is determined partially by the action of two type 3 secretion systems (T3SS), encoded by Salmonella pathogenicity island 1 (SPI-1) and SPI-2 (2, 3). The Salmonella T3SS act at different stages of infection and function to translocate a repertoire of bacterial effectors into the host cell (4). SPI-1 T3SS effectors mediate attachment to and invasion of the host cell, early biogenesis of the Salmonella-containing vacuole (SCV), and immune evasion. Following acidification of the SCV lumen, the SPI-2 T3SS is activated and translocates effectors across the vacuolar membrane. These contribute to immune evasion and facilitate SCV maturation and positioning near the microtubule-organizing center (MTOC) and Golgi apparatus, which contribute to bacterial replication (4, 5). SseF and SseG are two functionally related SPI-2 effectors that in epithelial cells maintain positioning of SCVs close to the MTOC/Golgi apparatus and enable the formation of a dense “microcolony” of SCVs. In the absence of SseF and SseG, SCVs disperse throughout the cytoplasm and intracellular bacterial replication and virulence are reduced (6–9).

Many pathogens, including *S*. Typhimurium, colonize the epithelium of the intestine (10, 11). The intestinal epithelium undergoes rapid self-renewal by the perpetual division of stem cells located in the intestinal crypts to maintain tissue homeostasis, a process that is accelerated in response to bacterial infection (12). Stem cells generate fast-cycling progenitor cells that undergo up to six rounds of cell division while migrating upward from the crypts to the tips of the villi, where the epithelial cells are eventually shed into the intestinal lumen (13). Epithelial renewal and shedding are thought to constitute a functional barrier against enteric bacteria, as infected enterocytes can be shed continuously and replaced (14). Many enteric bacteria have evolved mechanisms to counteract cell turnover and enhance intestinal colonization (12). For example, in the intestinal progenitor cells of the rabbit ileum, Shigella flexneri promotes cell cycle arrest in the G_2_/M phase of the cell cycle through the action of the T3SS effector IpaB (15). Other enteric bacteria alter the infected host cell cycle by secreting cyclomodulin toxins. Cyclomodulins constitute a class of toxins secreted by enteric bacteria that alter the infected host cell cycle. For example, cytolethal distending toxin secreted by Campylobacter jejuni activates a DNA damage signaling pathway and consequently triggers G_2_/M cell cycle arrest (16).

We previously reported that *S*. Typhimurium preferentially invades mitotic cells (17). In addition, it was shown recently that *S*. Typhimurium infection of epithelial cells induces cell cycle arrest in G_2_/M phase (18). In this study, we further investigated the impact of intracellular *S*. Typhimurium on the host cell cycle and found that a large proportion of infected cells became binucleated, due to a block at the terminal stage of cell division, cytokinesis, as a result of the formation of microcolonies by the effectors SseF and SseG. Our results suggest that *S*. Typhimurium infection induces binucleation due to a physical block at the forming cleavage furrow and cytokinetic bridge. Tetraploidy is known to have an aberrant effect on cell proliferation; consistent with this, we found an SseF/SseG-dependent reduction in bromodeoxyuridine (BrdU) incorporation in epithelial cell DNA in the mouse intestine during *S*. Typhimurium infection.

## MATERIALS AND METHODS

### Bacterial strains.

The wild-type *S*. Typhimurium strains used in this study were 12023, SL1344, and LT2 (NTCC). The mutant strains were *S*. Typhimurium 12023 *ssaV*::aphT (kanamycin [Kan]), *sseG*::aphT (Kan), *sseF*::aphT (Kan), and Δ*sseFsseG* (6). The pFPV25.1 (ampicillin [Amp]) plasmid was used for enhanced green fluorescent protein (EGFP) expression and the pFCcGI (Amp) plasmid for mCherry expression.

### *In vitro* infections.

HeLa (ECACC 93021013) cells were grown in Dulbecco's modified Eagle's medium (DMEM) (PAA Laboratories) and hTERT-RPE1 (ATCC CRL-4000) cells in DMEM–Ham F-12 medium (Sigma) with 0.25% sodium bicarbonate and 1 mM glutamine (Sigma). Both media were supplemented with 10% fetal calf serum (FCS) (PAA Laboratories). All (wild-type and mutant) bacterial strains were grown overnight in LB at 37°C, subsequently diluted 1:33 in 3 ml of LB, and grown until the culture reached an optical density at 600 nm (OD_600_) of 1.5 to 2.0. When appropriate, bacterial cultures were supplemented with kanamycin (50 µg/ml) or ampicillin (50 µg/ml) for selection. Bacteria were diluted in Earle's balanced salt solution (EBSS) (Gibco) and added to cells at a multiplicity of infection (MOI) of approximately 100 and incubated for 15 min. Cells were washed in phosphate-buffered saline (PBS) and incubated for 1 h in growth media with 100 μg/ml of gentamicin. The gentamicin concentration was subsequently decreased to 20 μg/ml for the remainder of the infection.

### Mouse infections.

Female C57BL/6 mice (B and K Universal Ltd., United Kingdom) (6 to 12 weeks of age) approximately 20 g in body weight were inoculated with approximately 6 × 10^7^ CFU/ml of late-exponential-phase bacteria by oral gavage. Mice were given 10 mg/ml of BrdU diluted in their drinking water for the duration of the experiment. At 120 h postinoculation, mice were sacrificed and the small intestines collected. Serial dilutions of the remaining bacterial solutions were prepared and plated onto LB agar plates to determine the exact bacterial CFU used for the oral gavage.

### Ethics statement.

Mouse experiments were conducted in accordance to European Directive 2010/63/EU regulations with approval from the Imperial College, London Animal Welfare and Ethical Review Body (ICL AWERB) under the Personal Project license of David Holden (license 70/7768).

### Immunofluorescence microscopy.

Cells were fixed with 3.7% paraformaldehyde (PFA) for 20 min at room temperature and washed with PBS, and the PFA was quenched with 1 mM NH_4_Cl for 30 min. Cells were incubated with antibodies or dyes diluted in PBS–10% horse serum–0.1% saponin for 1 h. The primary antibodies used were mouse anti-α-tubulin (Sigma), mouse anti-MPM-2 (Millipore), mouse anti-Incenp (Abcam), mouse anti-Aurora B (BD), rabbit anti-Survivin (Abcam), rabbit anti-kinesin-like protein 1 (anti-MKLP-1) (SC867) (Santa Cruz), and mouse anti-γ-tubulin (Sigma), and the dyes used were wheat germ agglutinin (WGA) (Invitrogen) and DRAQ5 (Biostatus). Coverslips were mounted using Aqua PolyMount (Polysciences Inc.). The total fluorescence signal (integrated density) of Incenp, Survivin, Aurora B, and MKLP-1 divided by the area of each individual cell was quantified using ImageJ. Samples were all imaged using a confocal laser scanning microscope (LSM510 or LSM710; Zeiss) with 405-, 488-, and 633-nm-wavelength excitation lasers and a 63× Plan-Apochromat 1.40-numerical-aperture (NA) 190-mm-working-distance (WD) oil or a 40× C-Apochromat 1.2-NA 280-mm-WD Water objective.

### Immunohistochemistry of mouse small intestines.

The small intestines from mice were divided into approximately 1-cm portions, and each portion was opened with a longitudinal cut. After several washes with PBS, intestines were fixed with 4% PFA for 2 h and then perfused with 10% sucrose. Intestinal sections were embedded in optimal cutting temperature (OCT) compound and frozen to −80°C. A cryostat was used to cut 5- to 7-μm-thick transverse intestine slices, which were mounted onto microscope slides. The slices were washed with PBS, incubated with 2 N HCl for 30 min, and washed three times with 0.1 M Na_2_B_4_O_7_ (pH 9). For BrdU and ZO-1 labeling, the slices were permeabilized with PBS–0.1% Triton X-100–10% horse serum for 30 min and then incubated with anti-BrdU (Abcam; Ab 1893) and anti-ZO-1 (Zymed Laboratories 33-9100), followed by anti-sheep Alexa Fluor 637 and anti-mouse Alexa Fluor 488 secondary antibodies (Invitrogen), respectively, or phalloidin (Invitrogen). Immunohistochemistry images were acquired in a blind manner for BrdU, as images were selected by DAPI (4′,6-diamidino-2-phenylindole) and ZO-1 staining. Samples were all imaged using a confocal laser scanning microscope (LSM510 or LSM710; Zeiss).

### Live imaging.

hTERT-RPE1-H2B-GFP cells (kindly provided by Laurent Sansregret, Francis Crick Institute, London) seeded onto 35-mm-diameter Matek dishes were infected with mCherry-wild-type *S*. Typhimurium 12023 at an MOI of 100. Image acquisition began at 8 h postinfection, and cells were selected on the basis of alignment of chromosomes on the metaphase plate. Images were acquired every 2 min for a period of 2 h using a Zeiss Axiovert 200 M microscope (Zeiss) with 488- and 633-nm-wavelength excitation lasers and a 63× Plan-Apochromat 1.4-NA 190-mm-WD oil objective, controlled by Volocity (Improvision).

### Flow cytometry.

Cells were trypsinized for 5 min, recovered in complete medium, and centrifuged at 200 × *g* for 5 min. Pellets were resuspended in 3.7% PFA–PBS for 20 min, following which cells were pelleted and PFA quenched with 50 mM NH_4_Cl for 30 min. Cells were centrifuged and then resuspended in 0.08% Triton X-100–PBS for 10 min. Cells were incubated with primary antibodies for 1 h and secondary antibodies for 40 min in PBS–10% horse serum. DNA was stained with propidium iodide–PBS–0.5 mg/ml RNase A or DRAQ5 for 10 min at room temperature. The small intestines were collected from mice infected as described above. After washing with PBS was performed, the intestines were incubated with Accutase (eBioscience) for at least 16 h at 4°C. The supernatant was collected and centrifuged for 5 min at 200 × *g*, and the cells were washed three times with PBS. BrdU labeling was performed as described above. All flow cytometry analyses were carried out on a two-laser, four-color FACSCalibur flow cytometer (BD Biosciences). Collected data were analyzed with FlowJo software version 7.6 (TreeStar).

### Statistical analysis.

Results shown are means ± standard errors of the means (SEM). Statistical testing was performed using Student's *t* test (continuous data, 2 groups [*, *P* < 0.05; **, *P* < 0.01; ***, *P* < 0.001; N.S, not statistically significant]).

## RESULTS

### Intracellular *S*. Typhimurium induces binucleation of host cells.

**FIG 1:**
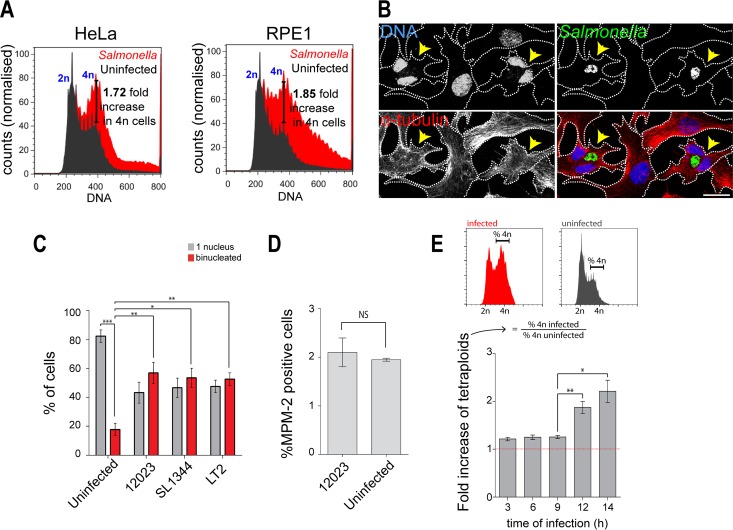
Intracellular *S*. Typhimurium infection leads to an accumulation of 4n/binucleated host cells. (A) Representative flow cytometry histograms of HeLa cells (left) and RPE1 cells (right) infected for 14 h with EGFP-*S*. Typhimurium 12023 and subsequently stained with DRAQ5 for cellular DNA content. The increase in the percentages of cells with cellular DNA content of 4n in the infected populations (red) compared to the uninfected populations (dark gray) is indicated. The indicated fold increase in 4n cells was calculated by dividing the percentage of infected 4n cells by the percentage of uninfected 4n cells from the same sample. (B) Immunofluorescence microscopy of RPE1 cells infected for 14 h with EGFP-*S*. Typhimurium 12023 (green), of DNA stained with DRAQ5 (blue), and of α-tubulin (red). Bar, 20 μm. (C) Percentages of RPE1 cells containing one or two nuclei, scored following fluorescence microscopy. Only nonoverlapping cells, as determined using cell membrane marker WGA, were analyzed. At least 400 cells were counted for each category in three independent experiments. (D) Flow cytometry analysis of RPE1 cells left uninfected or infected for 9 h with EGFP-*S*. Typhimurium 12023 and stained for DNA and MPM-2, a mitotic cell marker. Infected cells were gated by EGFP fluorescence. (E) Time course of the fold increase in 4n RPE1 cells infected with EGFP-*S*. Typhimurium, measured by flow cytometry analysis. As depicted in the histograms at the top of the panel, the fold increase of tetraploids was calculated by dividing the percentage of 4n infected cells by the percentage of 4n uninfected cells, from the same sample. (C, D, and E) Data are from three independent experiments where at least 30,000 cells were analyzed in each sample. *, *P* < 0.05; **, *P* < 0.01; ***, *P* < 0.001.

To investigate if intracellular *S*. Typhimurium interferes with the progression of the host cell cycle, we recorded the DNA profiles of asynchronous populations of HeLa cells and normal diploid human RPE1 cells infected for 14 h with wild-type *S*. Typhimurium strain 12023 constitutively expressing EGFP. Flow cytometry histograms of the cellular DNA content of infected cells showed an increase of approximately 1.7-fold to 1.9-fold in cells carrying two copies of the genome (“4n” DNA content, or tetraploid cells) compared to uninfected cells from the same samples (Fig. 1A; see also Fig. S1 in the supplemental material). This is in agreement with a previous study in which it was shown that Salmonella infection caused an arrest in the G_2_/M phase of the cell cycle (18). Tetraploid cells can arise from “mitotic slippage” or cytokinesis failure. “Mitotic slippage” describes cells that finish mitosis without progressing to anaphase or cytokinesis, due to an arrest of the spindle assembly checkpoint, resulting in cells with one tetraploid nucleus (19). In contrast, cytokinesis failure results in cells that contain two discrete nuclei, each with 2n DNA content (19). To distinguish between these possibilities, we used confocal fluorescence microscopy to image RPE1 cells that were infected for 14 h with wild-type *S*. Typhimurium strains. Strikingly, approximately 50% of the infected cells contained two nuclei, compared to 15% to 20% of the cells in the uninfected sample (Fig. 1B and C). This result was confirmed with two other wild-type strains of *S*. Typhimurium (SL1344 and LT2) in RPE1 and HeLa cells (Fig. 1C; see also Fig. S1), so all further experiments were conducted with strain 12023 in RPE1 cells. Unsurprisingly, the majority of binucleated cells infected with wild-type *S*. Typhimurium showed increased centrosome numbers compared to mononucleated cells (see Fig. S2A). We did not observe any differences between infected and uninfected cells in the mitotic index, as detected by the percentage of cells positive for MPM-2, a marker of mitosis, suggesting that infected cells do not arrest in mitosis (Fig. 1D). Taken together, these data suggest that *S*. Typhimurium induces binucleation of infected cells through a failure in cytokinesis.

### The SPI-2 T3SS effectors SseF and SseG cause binucleation of infected cells.

**FIG 2:**
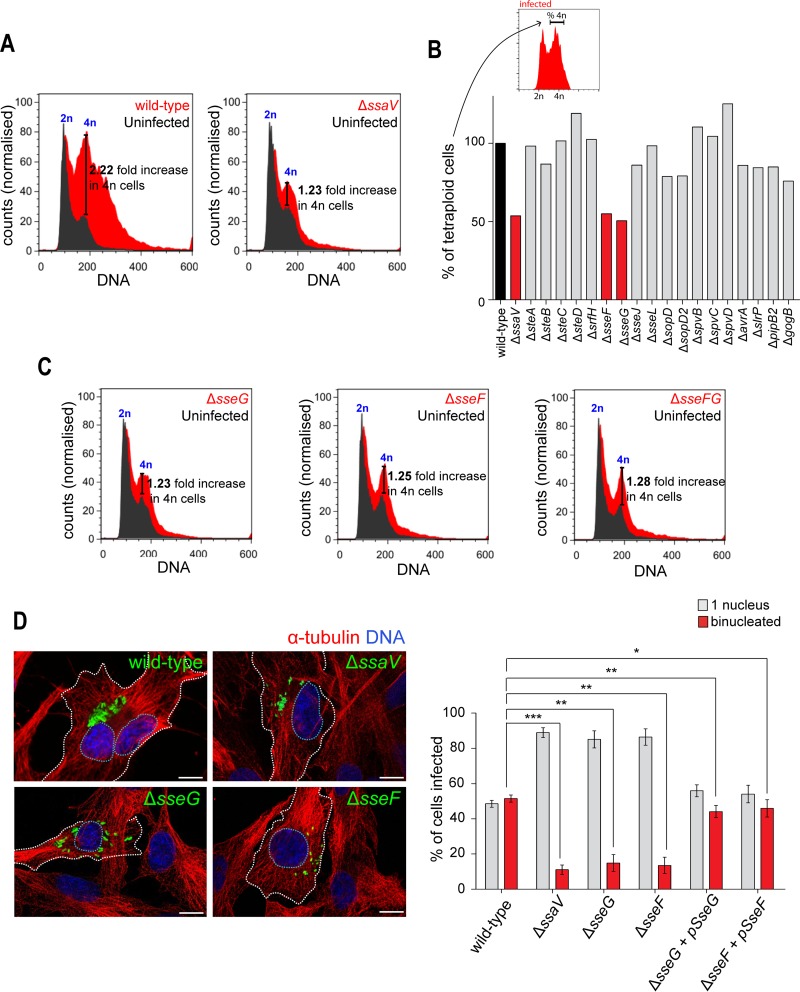
The SPI-2 T3SS effectors SseF and SseG of *S*. Typhimurium induce binucleation of host cells. (A) Representative flow cytometry DNA histograms showing RPE1 cells infected with wild-type *S*. Typhimurium (left, red) or the Δ*ssaV* mutant (right, red) compared with uninfected cells (dark gray). (B) Flow cytometry analysis of RPE1 cells infected with a panel of SPI-2 T3SS single-effector mutants. As depicted in the histogram at the top of the panel, the percentage of tetraploids was calculated by quantifying the number of cells with 4n DNA within the infected population and normalizing to the wild-type strain. The bars corresponding to the mutant strains considered to be positive hits are shown in red. At least 30,000 cells were analyzed in each sample. (C) Flow cytometry DNA histograms showing RPE1 cells infected with Salmonella mutant strain Δ*sseG*, Δ*sseF*, or Δ*sseFG* (red) and uninfected cells (dark gray). The increase in the percentages of infected cells with DNA content of 4n compared to the uninfected population is indicated. (D) Percentage of RPE1 cells containing one or two nuclei, scored following fluorescence microscopy (left panel). RPE1 cells were infected for 14 h with wild-type *S*. Typhimurium or the Δ*ssaV*, Δ*sseF* or Δ*sseG* mutant. Green, EGFP-*S*. Typhimurium; blue, host cell DNA stained with DRAQ5; red, α-tubulin. Dashed white lines highlight infected cells and nuclei. Bars represent 10 μm. At least 200 cells were counted for each category in three independent experiments. *, *P* < 0.05; **, *P* < 0.01; ***, *P* < 0.001. (A to C) The indicated fold increase in 4n cells was calculated by dividing the percentage of infected 4n cells by the percentage of uninfected 4n cells from the same sample. Data from flow cytometry analyses represent the results of at least three independent experiments, and at least 30,000 cells were counted in each sample for each experiment.

A significant increase in the number of tetraploid cells occurred from 12 h postinvasion onward (Fig. 1E), suggesting that bacterial replication and the action of the SPI-2 T3SS, but not of the SPI-1 T3SS, are required to perturb host cell division. A Δ*ssaV* mutant strain has an intracellular replication defect due to the absence of a functional SPI-2 T3SS (20). When cells were infected with the Δ*ssaV* mutant, there was an only moderate (1.23-fold) increase in numbers of cells containing 4n DNA relative to the uninfected population, in contrast to the increase of 2.22-fold in cells infected with wild-type bacteria (Fig. 2A). This suggests that Salmonella-induced host cell binucleation is dependent on the activity of one or more SPI-2 T3SS effectors. Of the 18 SPI-2 T3SS single-effector mutants subsequently tested, only the Δ*sseG* and Δ*sseF* mutants recapitulated the phenotype of the cells infected with the Δ*ssaV* mutant (Fig. 2B and C). A double Δ*sseFG* mutant did not further reduce the percentage of tetraploids (Fig. 2C), consistent with the known physical and functional interaction between SseF and SseG (6, 9, 21). In contrast to two other studies (18, 22), a Δ*spvB* mutant did not reduce the proportion of cells with 4n DNA content. Immunofluorescence analysis confirmed that the decrease in tetraploidy in cells infected with the Δ*ssaV*, Δ*sseF*, or Δ*sseG* mutant was due to a decrease in the level of binucleated cells, which was less than 20% of infected cells (Fig. 2D) and similar to the level in uninfected cells (Fig. 1C). Importantly, the lack of binucleation following infection with Δ*sseF* or Δ*sseG* mutants was complemented by the introduction of a low-copy-number plasmid carrying *sseF* or *sseG*, respectively (Fig. 2D). Therefore, SseF and SseG contribute to the formation of binucleated cells during *S*. Typhimurium infection.

### Bacterial microcolonies obstruct the cleavage furrow of diving cells.

**FIG 3:**
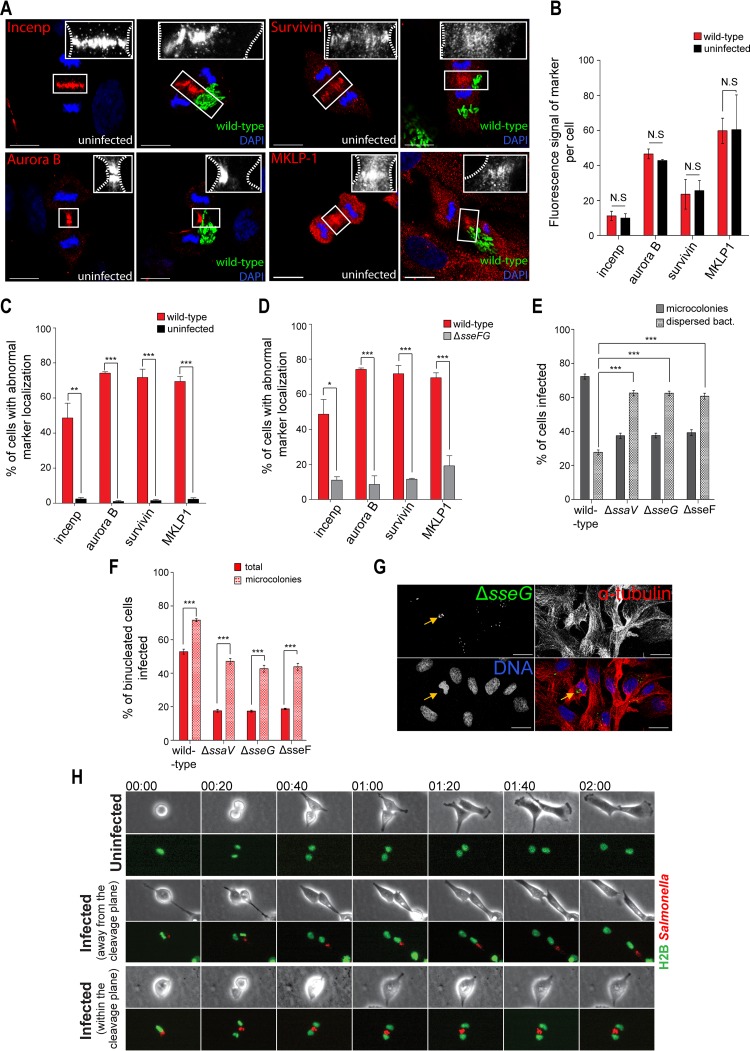
The microcolony formed by *S*. Typhimurium physically obstructs the cleavage furrow during abscission. (A) Representative images by immunofluorescence microscopy of uninfected RPE1 or cells infected with EGFP-expressing Salmonella (green), immunostained for Incenp (top left), Survivin (top right), Aurora B (bottom left), or MKLP-1 (bottom right) shown in red or white (zoom); DNA is shown in blue. Bars represent 20 μm. (B) Total fluorescence signal of Incenp, Survivin, Aurora B, and MKLP-1 with or without infection with wild-type Salmonella. (C) Percentage of RPE1 cells with mislocalization of Incenp, Survivin, Aurora B, and MKLP-1 in uninfected cells or wild-type-infected cells. (D) Percentage of RPE1 cells with mislocalization of Incenp, Survivin, Aurora B, and MKLP-1 in wild-type-infected cells or Δ*sseFG* mutant-infected cells. (E) Percentage of RPE1-infected cells with a microcolony or with dispersed bacteria, scored following fluorescence microscopy. (F) Percentage of binucleated RPE1 cells in all infected cells or only in cells containing a microcolony following fluorescence microscopy. (G) Immunofluorescence microscopy image of RPE1 cells infected with the EGFP-Δ*sseG* mutant strain (green). Red, α-tubulin; blue, DNA. The arrows point to an infected binucleated cell with a microcolony. Bars represent 20 μm. (H) Time-lapse microscopy of cell division in RPE1 cells stably expressing histone 2B (H2B) fused with EGFP (green)- and mCherry (red)-expressing *S*. Typhimurium showing an uninfected cell (first panel), an infected cell where the microcolony localized away from the cleavage plane (second panel), and an infected cell where the microcolony localized within the cleavage plane, leading to failure in abscission and subsequent binucleation (third panel). Time shown in hours:minutes. (B to F) At least 400 cells were counted in three independent experiments *, *P* < 0.05; **, *P* < 0.01; ***, *P* < 0.001; N.S, not statistically significant.

To further analyze if there was a cytokinesis defect in cells infected with Salmonella, we examined the localization of three members of the chromosomal passenger complex (CPC), Incenp, Survivin, and Aurora B, as a measure for correct formation of the cleavage furrow. The CPC localizes to the cleavage furrow, where it has a crucial role in furrow formation and function (23). In addition to members of the CPC, we also examined the localization of mitotic kinesin-like protein 1 (MKLP-1), a member of the centralspindlin complex that contributes to cleavage furrow ingression and facilitates recruitment of the CPC and proteins that control abscission (24–26). While no differences in the total levels of Incenp, Survivin, Aurora B, or MKLP-1 were detected upon infection (Fig. 3A and B), approximately 50% of the cells infected with the wild-type strain showed abnormal localization of Incenp and approximately 70% showed abnormal localization of Aurora B, Survivin, and MKLP-1 at the cleavage furrow, in comparison to less than 3% of uninfected cells (Fig. 3A and C). Only approximately 10% of cells infected with the Δ*sseFG* mutant showed abnormal localization of Incenp, Aurora B, and Survivin and approximately 20% abnormal localization of MKLP-1 (Fig. 3D). When oriented within the cleavage plane, foreign bodies such as latex beads, Chlamydia inclusions, phagocytosed dead cells, or asbestos fibers can disrupt cytokinesis by perturbing cleavage furrow formation and physically blocking abscission (27–30). *S*. Typhimurium mutant strains lacking either a functional SPI-2 T3SS (Δ*ssaV*) or either SseF or SseG form few MTOC-associated microcolonies; instead, the SCVs have a tendency to scatter throughout the cells (8). We scored infected cells based on the presence of a microcolony (defined as a tight cluster of SCVs near the MTOC) or scattered SCVs (Fig. 3E) and then quantified the number of binucleated cells in these subpopulations. Strikingly, where infected cells harbored a microcolony, we found a high enrichment of binucleated cells for all strains, including the mutants (Fig. 3F). Therefore, the presence of a dense microcolony is likely to cause most of the cytokinesis failure events during Salmonella infection by physically blocking abscission. Nevertheless, by comparing wild-type and mutant Salmonella results, it was apparent that the presence of a microcolony did not completely account for binucleation, as the level of binucleation in Δ*ssaV*, Δ*sseF*, and Δ*sseG* mutant-infected cells that harbored a microcolony was not as high as in wild-type-infected cells with a microcolony. Given that Δ*ssaV*, Δ*sseF*, and Δ*sseG* mutants all have significant replication defects in epithelial cells, the number of intracellular bacteria and hence the size of the microcolony could also contribute to the block in cytokinesis (Fig. 3F and G) (7, 9, 20). SseF/SseG-mediated microtubule bundling has been observed during Salmonella infection (31). There was no apparent microtubule bundling in RPE1 cells infected with wild-type Salmonella at 14 h compared to uninfected and Δ*sseF*, Δ*sseG*, and Δ*ssaV* mutant-infected cells (see Fig. S2B in the supplemental material), suggesting that microtubule bundling is a cell type-dependent phenotype and is unlikely to contribute to the cytokinesis defect that we observed (31). We then conducted live imaging of RPE1 cells stably expressing histone 2B and GFP (H2B-GFP) to observe cell division in uninfected and infected cells. Cell rounding, chromosome condensation, and lining up of chromosomes at the metaphase plate were unaffected in infected cells (Fig. 3H; see also Movies S1 to S3 in the supplemental material). Normal cell division occurred in infected cells if the microcolony was not located within the cleavage furrow (middle panel, Fig. 3H; see also Movie S2). However, when the microcolony was positioned between the two separated chromosome masses during telophase, the cleavage furrow formed and collapsed, generating a binucleated cell (bottom panel, Fig. 3H; see also Movie S3). Taken together, these data suggest that the presence of *S*. Typhimurium microcolonies within the cleavage furrow acts as a physical barrier that displaces essential proteins for abscission, often resulting in failure during cytokinesis and subsequent binucleation of the host cell.

### *S*. Typhimurium infection inhibits cell turnover at the small intestine.

**FIG 4:**
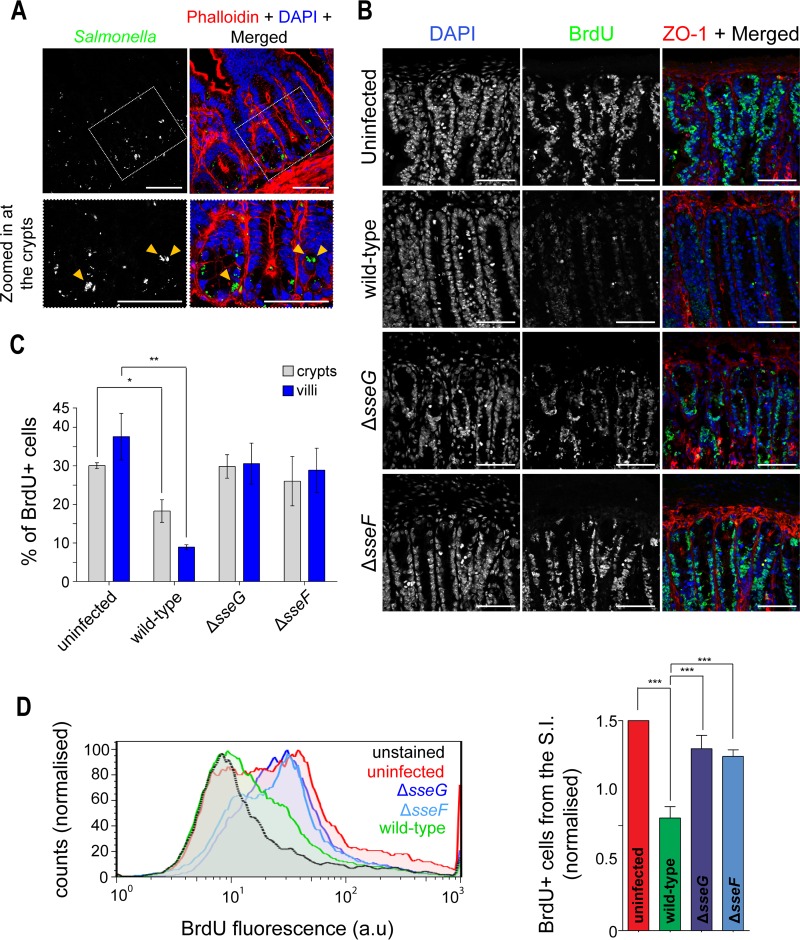
*S*. Typhimurium impairs cell proliferation at the mouse small intestine in an SseF- and SseG-dependent manner. (A) Histochemistry images from slices of the small intestines of mice infected with mCherry-wild-type *S*. Typhimurium 12023 120 h after oral inoculation. *S*. Typhimurium is shown in green, DNA in blue, and actin in red. Zoomed images are shown in the bottom panels. Arrowheads point at infected cells with microcolonies. Bars represent 50 μm. (B) Immunohistochemistry images from slices of the small intestines of uninfected mice (first panel) or of mice infected with wild-type *S*. Typhimurium (second panel), the Δ*sseG* mutant (third panel), or the Δ*sseF* mutant (fourth panel) and given BrdU for 120 h. DNA is shown in blue, BrdU in green, and ZO-1 in red. Bars represent 50 μm. (C) Percentage of BrdU-positive cells from the crypts or villi left uninfected or infected with wild-type, Δ*sseG*, or Δ*sseF* *S*. Typhimurium, quantified from immunofluorescence microscopy. One mouse was infected per strain in four independent experiments. At least 100 cells were counted per strain in each experiment. (D) Flow cytometry histograms showing BrdU fluorescence in cells isolated from the small intestines (S.I.) of mice left uninfected or infected with wild-type, Δ*sseG*, or Δ*sseF* mutant *S*. Typhimurium (left) and the corresponding quantification of BrdU-positive cells normalized to the uninfected sample (right). An unstained sample was used to provide the background signal used as the threshold to distinguish BrdU-positive from BrdU-negative cells. At least 30,000 cells were counted per sample. (C and D) One mouse was sacrificed per condition, and at least 4 independent experiments were performed. *, *P* < 0.05; **, *P* < 0.01; ***, *P* < 0.001.

The inevitable consequence of tetraploidy is an aberrant effect on subsequent rounds of cell division and overall proliferation, as a result of an arrest in the following cell cycle or cell death or a development of chromosome instability and aneuploidy (19, 32–34). To measure the effect of SseF and SseG on cell proliferation in the epithelium of the small intestine, we infected mice by oral gavage for 120 h with wild-type or mutant *S*. Typhimurium strains constitutively expressing mCherry, as this fluorescent protein is more photostable *in vivo* than GFP (35). Oral inoculation of mice with *S*. Typhimurium induces a systemic disease with successful colonization of the epithelium of the small intestine, followed by invasion of the lamina propria and seeding of the liver, spleen, and gallbladder (36). Infected mice were sacrificed and their small intestines removed and sectioned for immunohistochemistry analysis. In agreement with other studies (37, 38), we found that *S*. Typhimurium can access not only the villi but also the crypts of the small intestine (Fig. 4A—Salmonella microcolonies are indicated by the yellow arrowheads). Resident stem cells at the intestinal crypts give rise to fast-proliferating cells that migrate upward to regenerate the layer of enterocytes in the villi, in a continuous and well-characterized process (39). We determined if *S*. Typhimurium infection impaired the rate of overall cell proliferation (and, indirectly, the cell cycle) in the small intestine, by adding 5-bromo-2′-deoxyuridine (BrdU) to the drinking water of mice during infection. In uninfected mice, the proportions of BrdU-positive nuclei observed by confocal microscopy were approximately 30% of cells within the crypts and 35% within the villi. Similar percentages were found for both the Δ*sseF* and the Δ*sseG* mutant strains (∼30% for both mutants in the crypts and villi) (Fig. 4B and C). In contrast, the proportions of BrdU-positive nuclei in the crypts in wild-type-infected samples were approximately 15% and less than 10% at the villi (Fig. 4B and C). These results were confirmed by flow cytometry, where the proportions of BrdU-positive cells extracted from the small intestine of wild-type-infected mice were approximately 45% less than the amount seen with Δ*sseF-* and *ΔsseG*-infected mice and 55% less than the amount seen with the uninfected control. (Fig. 4D). Therefore, this set of data indicates that *S*. Typhimurium impairs cell turnover in the small intestine in an SseF/SseG-dependent manner.

## DISCUSSION

Analysis of infected RPE1 cells showed that the presence of intracellular *S*. Typhimurium led to an accumulation of binucleated cells after 12 h of infection (Fig. 1). This accumulation depended on two SPI-2 T3SS effectors, SseF and SseG (Fig. 2), which promote the formation of stable bacterial microcolonies in the vicinity of the Golgi network (6–9). Live-cell imaging and immunofluorescence analysis revealed that binucleation often occurred when a microcolony of SCVs was observed and localized within the cleavage furrow during cytokinesis, which strongly indicates that the *S*. Typhimurium microcolony induced binucleation (Fig. 3).

Chlamydia trachomatis inclusions, phagocytosed synthetic fibers, phagocytosed cells, and latex beads have all been shown to physically block furrow ingression and cytokinesis and lead to cell binucleation (27, 29, 30, 40). As infection progresses, SCVs move toward the Golgi apparatus and, following bacterial replication, form a dense microcolony. A functional SPI-2 T3SS is important for SCV positioning at the Golgi apparatus and microcolony formation, due to the action of SseF and SseG (6–9). Salmonella-induced binucleation also depended on SseF and SseG. Previous work has indicated that expression of SseF and SseG during infection of HeLa cells at 16 h can cause the bundling of microtubules emanating from the microcolony of SCVs by an unknown mechanism (31). Bundled microtubules could conceivably interfere with cytokinesis due to the fundamental importance of dynamic microtubule networks in cytokinesis and all stages of mitosis, or bundled microtubules could represent a physical block to the forming cleavage furrow. We did not observe bundled microtubules in infected RPE1 cells (see Fig. S2B in the supplemental material). Therefore, this phenomenon, which in HeLa cells is dependent on SseF and SseG, does not appear to contribute to cytokinesis failure in RPE1 cells (31). Indeed, cytokinesis failure is unlikely to be due to direct mechanistic action of SseF or SseG, as binucleation was still observed in the absence of these effectors in the minority of cells that formed dense microcolonies. In addition, cell abscission failed and the CPC and MLKP-1 were abnormally localized in a microcolony observed within the cleavage furrow (Fig. 3). Therefore, the most likely explanation is that the microcolony of SCVs leads to binucleation of Salmonella-infected cells by causing a physical block at the cleavage furrow and preventing the completion of cytokinesis.

It is well established that tetraploidy or cytokinesis failure negatively affects subsequent cell proliferation (32–34, 41, 42). To investigate intestinal cell proliferation *in vivo*, we measured BrdU incorporation in DNA of intestinal cells of mice infected with wild-type or mutant strains. We found that *S*. Typhimurium infection impaired cell proliferation at the small intestine, in an SseF/SseG-dependent fashion (Fig. 4). Since BrdU would be incorporated only into the DNA of proliferating cells, we conclude that the effects of SseF and SseG on the host cell cycle might also occur *in vivo*. However, reduced proliferation could also be due to increased apoptosis and infection-induced inflammation (43). The replication of Δ*sseF* and Δ*sseG* mutant strains is compromised relative to that of wild-type Salmonella in mouse models of infection (6). Therefore, the differences in the levels of intestinal cell proliferation detected after infection with wild-type Salmonella versus the Δ*sseF* or Δ*sseG* mutants could also be due to their intracellular replication defect. This in turn could lead to reduced inflammation and cell damage. Further work studying intestinal morphology and levels of inflammation and gut bacterial burden might help to clarify the specific effects of bacterial replication and inflammation and host cell cycle failure on intestinal cell turnover.

Nevertheless, our work gives a strong indication that *S*. Typhimurium infection reduces the propensity for the gut epithelium to proliferate. The self-renewal of the gut epithelium is thought to be an important host defense mechanism to eliminate infected cells and therefore to limit persistent bacterial colonization. Some bacterial pathogens, such as enteropathogenic Escherichia coli (EPEC) or Shigella, counteract the rapid epithelial turnover to maintain enterocytes as a replicative niche. Some EPEC strains deliver the protein Cif into host cells, which blocks the host cell cycle by causing both G_1_/S and G_2_/M arrests (44, 45). Interestingly, Shigella seems to be able to manipulate epithelial cell turnover by directly accessing the progenitor cells in the crypts via the translocation of the T3SS effector IpaB, which arrests cell cycle progression by direct binding to Mad2L2, an anaphase-promoting complex inhibitor (15). Similarly, we show that *S*. Typhimurium accesses the epithelial crypts in the mouse model of infection (Fig. 4A) (38). By blocking cell proliferation in the intestinal crypts, *S*. Typhimurium could delay epithelial cell turnover to facilitate bacterial colonization.

## Supplementary Material

Supplemental material
