# Supplementary material for: Clustered Intracellular Salmonella enterica Serovar Typhimurium Blocks Host Cell Cytokinesis
Source: Infect Immun. 2016 Jun 23;84(7):2149–58. doi: 10.1128/IAI.00062-16 (PMC4936369; doi:10.1128/IAI.00062-16)
Supplement: Supplemental material [file supp_84_7_2149__index.html]

Supplemental material 

# Clustered Intracellular Salmonella enterica Serovar Typhimurium Blocks Host Cell Cytokinesis

## Supplemental material

- Supplemental file 1 -

  Fig. S1. Intracellular *Salmonella* induces an increase of cells with 4n DNA. Fig. S2. Binucleated cells induced by intracellular *S.* Typhimurium carry two or three centrosomes. Legends for Movies S1 to S3.

  PDF, 1.5M
- Supplemental file 2 -

  Movie S1. Time-lapse microscopy of cell division in an uninfected RPE1 cell stably expressing histone 2B fused with EGFP.

  MOV, 1.6M
- Supplemental file 3 -

  Movie S2. Time-lapse microscopy of successful cell division in an RPE1 cell infected with mCherry-expressing *S.* Typhimurium stably expressing histone 2B fused with EGFP.

  MOV, 3.5M
- Supplemental file 4 -

  Movie S3. Time-lapse microscopy of unsuccessful cell division in an RPE1 cell infected with mCherry-expressing *S.* Typhimurium stably expressing histone 2B fused with EGFP.

  MOV, 2.3M
