# Supplementary material for: Clustered Intracellular Salmonella enterica Serovar Typhimurium Blocks Host Cell Cytokinesis
Source: Infect Immun. 2016 Jun 23;84(7):2149–58. doi: 10.1128/IAI.00062-16 (PMC4936369; doi:10.1128/IAI.00062-16)
Supplement: Supplemental material [file IAI.00062-16_zii999091759so1.pdf]

Supporting Information

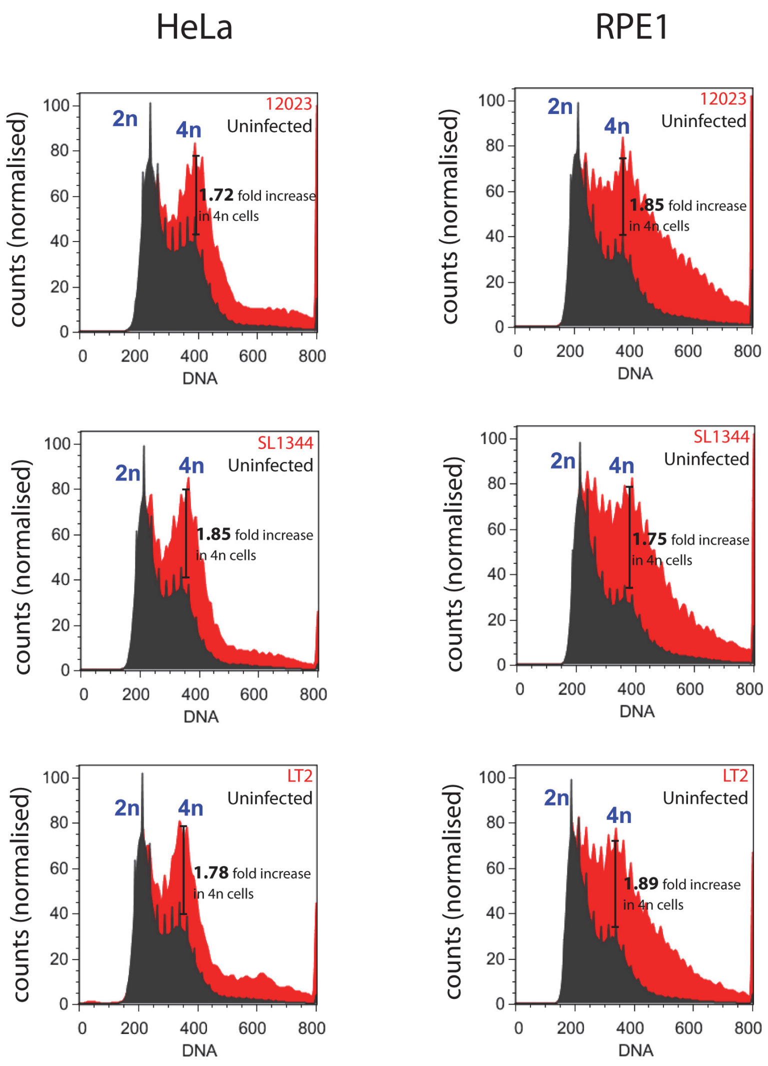

Figure S1. Intracellular *Salmonella* induces an increase of cells with 4n DNA. Asynchronous populations of HeLa cells (left) and RPE1 cells (right) were infected for 14 h with EGFP-expressing wild-type *Salmonella* strain 12023, SL1344 and LT2. Host cellular DNA was stained with DRAQ5. The increase in the percentages of infected cells (red) with DNA content of 4n compared to the uninfected population (dark grey) is indicated. The indicated fold increase in 4n cells was calculated by dividing the percentage of infected 4n cells by the percentage of uninfected 4n cells from the same sample. At least three

independent experiments were made and for each experiment at least 30,000 cells were analysed in each sample.

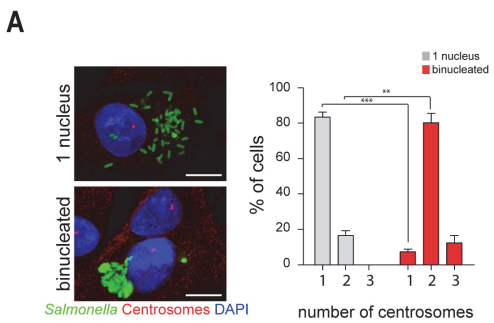

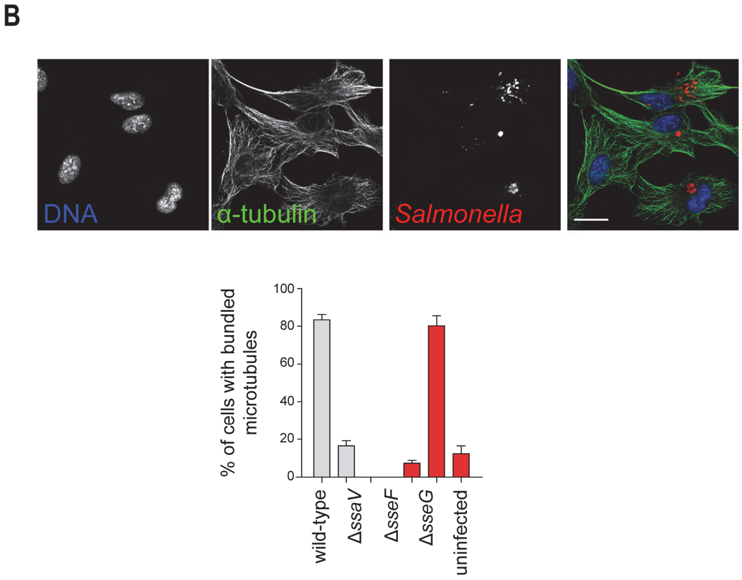

Figure S2. Binucleated cells induced by intracellular *S.* Typhimurium carry 2 or 3 centrosomes. (A) Confocal microscopy of RPE1 cells infected for 14 h with EGFP-S. Typhimurium 12023 (green) showing the number of centrosomes in infected cells; DAPI (blue); γ-tubulin (red). Bars represent 20 μm (left). The percentage of mono or bi-nucleated cells were scored for the number of centrosomes per cell (right). Approximately 200 cells were counted across four independent experiments. $^{**}P<0.01$, $^{***}P<0.001$. **(B)** Confocal microscopy of RPE1 cells infected for 14 h with mCherry-S. Typhimurium 12023 (red), and stained for DAPI (blue) and α-tubulin (green). Bars represent 20 μm (left). The percentage of cells with bundled microtubules were quantified

(below). Approximately 100 cells were counted in each of 2 independent experiments.

Movie S1: Time-lapse microscopy of cell division in an uninfected RPE1 cell stably expressing Histone 2B (H2B) fused with EGFP (green). Bright field images were acquired every 2 minutes and fluorescent images every 20 minutes for a period of 2 hours using a Zeiss Axiovert 200 M microscope (Zeiss) controlled by Volocity (Improvision). Time shown in hh:mm.

Movie S2: Time-lapse microscopy of successful cell division in a RPE1 cell infected with mCherry-expressing *S*. Typhimurium (red) stably expressing Histone 2B (H2B) fused with EGFP (green). Image acquisition began at 8 h post-infection. Bright field images were acquired every 2 minutes and fluorescent images every 20 minutes for a period of 2 hours using a Zeiss Axiovert 200 M microscope (Zeiss) controlled by Volocity (Improvision). Time shown in hh:mm.

Movie S3: Time-lapse microscopy of unsuccessful cell division in a RPE1 cell infected with mCherry-expressing *S*. Typhimurium (red) stably expressing Histone 2B (H2B) fused with EGFP (green). Image acquisition began at 8 h post-infection. Bright field images were acquired every 2 minutes and fluorescent images every 20 minutes for a period of 2 hours using a Zeiss Axiovert 200 M microscope (Zeiss) controlled by Volocity (Improvision). Time shown in hh:mm.
